# Compensation for Changing Motor Uncertainty

**DOI:** 10.1371/journal.pcbi.1000982

**Published:** 2010-11-04

**Authors:** Todd E. Hudson, Hadley Tassinari, Michael S. Landy

**Affiliations:** 1 Department of Psychology, New York University, New York, New York, United States of America; 2 Center for Neural Science, New York University, New York, New York, United States of America; Northwestern University, United States of America

## Abstract

When movement outcome differs consistently from the intended movement, errors are used to correct subsequent movements (e.g., adaptation to displacing prisms or force fields) by updating an internal model of motor and/or sensory systems. Here, we examine changes to an internal model of the motor system under changes in the variance structure of movement errors lacking an overall bias. We introduced a horizontal visuomotor perturbation to change the statistical distribution of movement errors anisotropically, while monetary gains/losses were awarded based on movement outcomes. We derive predictions for simulated movement planners, each differing in its internal model of the motor system. We find that humans optimally respond to the overall change in error magnitude, but ignore the anisotropy of the error distribution. Through comparison with simulated movement planners, we found that aimpoints corresponded quantitatively to an ideal movement planner that updates a strictly isotropic (circular) internal model of the error distribution. Aimpoints were planned in a manner that ignored the direction-dependence of error magnitudes, despite the continuous availability of unambiguous information regarding the anisotropic distribution of actual motor errors.

## Author Summary

To plan effective movements of the limbs, the human motor system must keep track of certain parameters: Obvious examples are the lengths and masses of to-be-controlled limb segments. In addition, the nervous system tracks its own motor outcome noise, which is important for selecting among movement plans where there are substantial costs associated with movement inaccuracies (e.g., reaching past a glass of red wine on a cluttered dinner table). Here, we introduce a change in motor noise in a reaching task: reaches were perturbed unpredictably by activating a reflex that introduced unplanned horizontal arm motion at the ends of reaches. We show that the motor system updates an internal model of the overall increase in motor noise induced by this reflex perturbation, but fails to represent the anisotropic component of the noise. This result is consistent with current theories of motor planning and control in which reach magnitudes and directions are represented independently, because a system that updates only a circular representation of recent motor errors is equivalent to a system that monitors only the magnitudes of recent errors, and ignores their directions.

## Introduction

The motor system is exquisitely sensitive to perturbation. The ability to sense a discrepancy between planned and executed movement and respond accordingly is one of the hallmarks of motor learning [1], [2], [3], [4]. Here, we are concerned with the nature of the error signal used to update future movement plans when the result of a movement does not match the intended outcome. Of course there is an infinite number of statistics of the error signal that the CNS might use to update future motor plans, ranging from a running average of recent errors, to *n* ^th^-order moments of the distribution of past errors. We are interested in exploring the limits of what statistics can be modeled by the nervous system.

Previous work has focused on neuromotor corrections to imposed bias, where corrective responses are found opposite to the direction of previous errors, and proportional to prior error extents [5], [6], [7]. This work supports motor learning models in which future motor plans incorporate an inverse of the command that would have produced the previous error. This deterministic model of motor learning suggests that errors from past movements are subtracted off of future motor plans. Such models can be traced at least to Helmholtz [8], who used this type of model to describe perceptual constancy following eye movements.

However, these deterministic models fail to recognize that the CNS can neither simply “read off” a motor error from noisy sensory signals, nor can it produce identical motor outcomes with repetitions of motor commands. The relationships between sensory signal and motor error, and between motor command and motor outcome, must be inferred; those inferences are far from certain. Recognizing this, current research has examined the role of uncertainty in motor learning [e.g.], [ 9,10]. For example, Sheidt et al. [10] added a stochastic element to an average force field and found that subjects adapted to the uncertain field strength by tracking its expectation over recent errors. Here, we are interested in the response to changes in motor uncertainty, and ask whether these responses result from updating an internal model of motor variance; and if so, which aspects of the variance structure of the uncertain error signal are modeled.

In these studies, we increased motor noise anisotropically by stimulating a reflexive motor response known to occur when reaching in the presence of horizontal visual-field motion, or ‘drift’ [11]. From trial to trial observers were shown leftward motion, rightward motion or a static stimulus, in random order. The motion, if present, began at the halfway-point of the reach, and resulted in a perturbation of the reach in the direction of the visual motion. Subjects could not plan in advance for any particular drift condition since these were randomly intermixed, nor could they compensate for the drift online because the timing of the reach and drift-onset insured that reaches were completed before feedback correction was possible [11]. Because this reflexive manual following response (MFR) affects only the horizontal component of a reach, it was possible to test which aspects of the new, anisotropic distribution of motor errors was modeled by the CNS.

**Figure 1:**
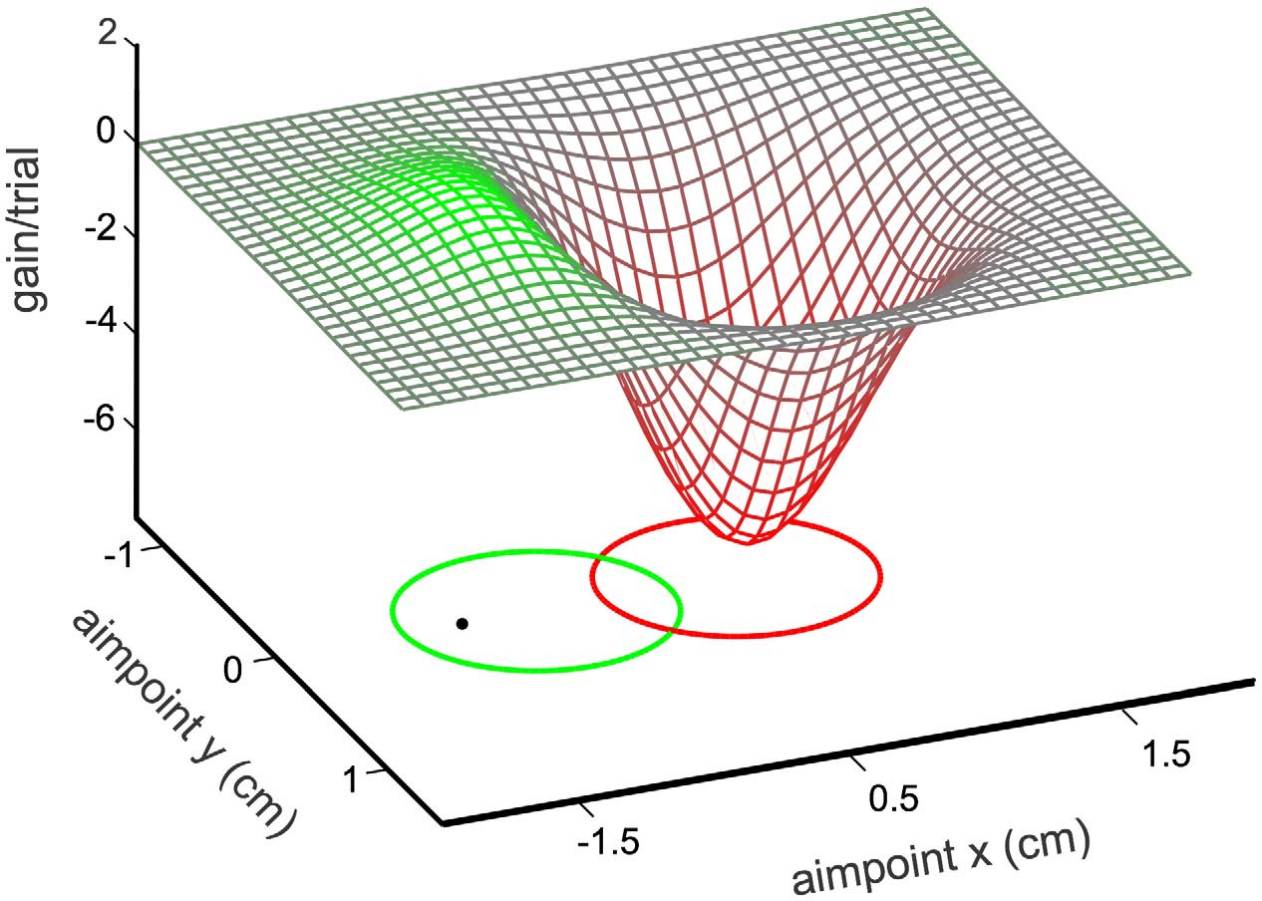
Example stimulus with superimposed expected gain landscape. In our task, subjects made rapid pointing movements at a frontoparallel screen to hit a green target circle while avoiding an overlapping red penalty circle (here shown on the *x-y* plane). For this illustration, hits on the green circle result in a reward of 1 point and hits on the red circle yield a penalty of 5 points. This figure shows the expected gain per trial associated with different aimpoints for a simulated subject with a circular Gaussian movement uncertainty of 3 mm standard deviation. The optimal aimpoint maximizing expected gain (MEG aimpoint) is the filled circle marked within the green target region.

We test for changes in the internal representation of motor noise by monitoring changes in reach plans toward visible targets, which depend on the details of the information available to the CNS concerning motor uncertainty. In these experiments, successful reaches to targets earn subjects a monetary bonus; reaches that instead intersect a neighboring region of the screen induce a monetary loss (Fig. 1). In two sessions, each beginning with reaches to targets without penalties, subjects learn their natural and perturbed (anisotropic) noise distributions, and then respond to target-penalty pairs later in the session, allowing us to assess their internal representation of motor uncertainty.

Our results indicate that the CNS updates a strictly circular internal model of motor variance, even when the distribution of actual errors is anisotropic. This result is consistent with recent psychophysical and neurophysiological results [3], [6], [12], [13] indicating independent encoding of the directions and extents of movement errors, because a system that updates only a circular internal representation of errors is equivalent to a system that monitors only the magnitudes of those errors, ignoring their directions.

## Results

We are interested in how the CNS compensates for changing motor uncertainty. We consider two possibilities: compensation is mediated via a hill-climbing mechanism using incremental corrections based on past errors [14], or alternatively by updating an internal model of motor variance. In the latter case, we are interested in which aspects of the variance structure of the uncertain error signal are modeled. For the case of a hill-climbing mechanism, incremental correction can produce anisotropic adaptation when sensory or motor uncertainty are anisotropically perturbed, such as observed in previous work demonstrating adaptation to anisotropically increased sensory [15] and motor [16; see Discussion for further detail] errors. Because compensations within a hill-climbing mechanism are based only on incremental correction of errors rather than an estimate of parameters describing the underlying motor system, there is no requirement that any internal model be used or formed.

### Reach endpoints

**Figure 2:**
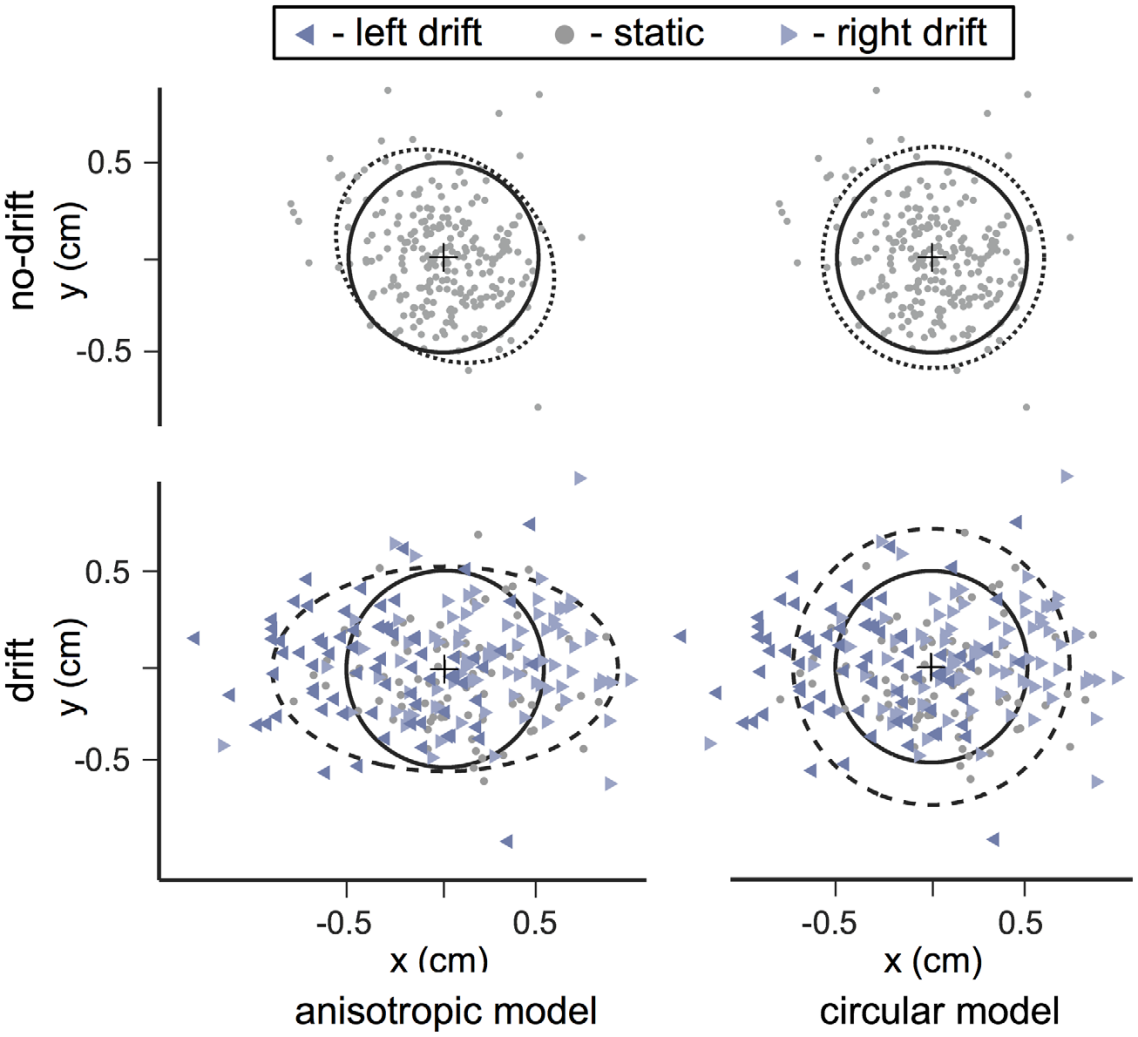
Effects of movement perturbation on movement endpoints. Movement endpoints are shown for subject S4 for zero-penalty trials aiming at the green target circle. Solid circles represent the 1 cm target region. Upper row: no-drift session. Lower row: drift session. Left column: dotted and dashed curves are 2-SD covariance ellipses. Right column: Dotted and dashed curves are 2-SD circles of an isotropic Gaussian fit to the data. Different symbols indicate the drift condition.

Fig. 2 shows reach endpoints from the zero-penalty blocks of the unperturbed or ‘no-drift’ (top row) and perturbed or ‘drift’ (bottom row) sessions from subject S4 (results from other subjects' drift sessions are available in Supplementary Figure S1). For all four plots, the solid circle represents the 1 cm diameter target, while dashed and dotted ellipses represent the covariance ellipse (drift and no-drift, respectively) in the left column, and the dashed and dotted circles represent circular Gaussian fits to the same endpoint data (right column). Clearly, when there was no penalty, subjects aimed at the center of the target circle. Bias (in any direction) from the target center never exceeded 2 mm for any subject during zero-penalty trials. The average bias in the (task-relevant) horizontal and vertical directions across all subjects was less than 1 mm. For each subject, the 95% confidence interval for the horizontal and vertical biases always overlapped zero. We conclude that no mean endpoint was significantly different from the center of the target for any subject or condition during zero-penalty trials. In the no-drift session, endpoint variance was nearly identical for the horizontal and vertical directions. During the drift session, unpredictable MFR perturbations substantially increased horizontal endpoint variance, but did not alter vertical variance (Table 1). Endpoints from leftward drift are displaced to the left, and those from rightward drift are displaced rightward, as expected.

**Table 1:** Comparisons of endpoint variance.

Test Type	Subject	S1	S2	S3	S4	S5	S6	S7	S8	S9
vertical drift	*F*(239,239)	0.87	1.10	1.23	0.95	0.86	1.26	1.05	0.68	1.15
vs	*p*-value	0.86	0.235	0.055	0.646	0.873	0.038	0.365	0.999	0.134
vertical no-drift	*evidence (dB)*	−19.7	−9.8	133	−3.4	−12.7	−0.23	−7.62	−20.0	−2.9
horizontal drift	*F*(239,239)	3.56	3.07	3.15	2.78	3.51	3.17	3.77	2.47	0.93
vs horizontal	p-value	<.001	<.001	<.001	<.001	<.001	<.001	<.001	<.001	0.707
no-drift	*evidence (dB)*	509	196	936	309	172.6	145.8	198.0	88.5	−15.4
horizontal	*F*(239,239)	0.66	0.90	1.03	0.96	0.83	0.67	0.60	0.56	0.78
vs vertical	p-value	0.999	0.789	0.416	0.606	0.926	0.999	0.999	0.999	0.967
(no-drift)	*evidence (dB)*	−19.5	−23.1	−26.5	−24.5	−13.5	−16.7	−18.3	−22.8	−28.0
horizontal	*F*(239,239)	2.73	2.52	2.63	2.82	3.33	1.68	2.17	2.07	0.635
vs vertical	p-value	<.001	<.001	<.001	<.001	<.001	<.001	<.001	<.001	0.999
(drift)	evidence (dB)	151	25.0	266	62.5	163.5	24.8	64.6	54.7	−13.1

*F*- and *p*-values are given for comparisons of the horizontal and vertical variances in the drift- and no-drift conditions (for zero-penalty trials only). No Bonferroni corrections were applied, although no conclusions would be changed by applying corrections. For the first and third comparison, positive evidence indicates support for a model in which the two variances are unequal vs one that assumes they are equal. For the second comparison, positive evidence supports a model that assumes horizontal variance is greater in the drift trials than in the no-drift trials (versus a model that assumes equal variances). In the fourth comparison, positive evidence values support a model that assumes horizontal variance is greater than vertical variance (versus a model that assumes equal variances).

**Figure 3 pcbi-1000982-g003:**
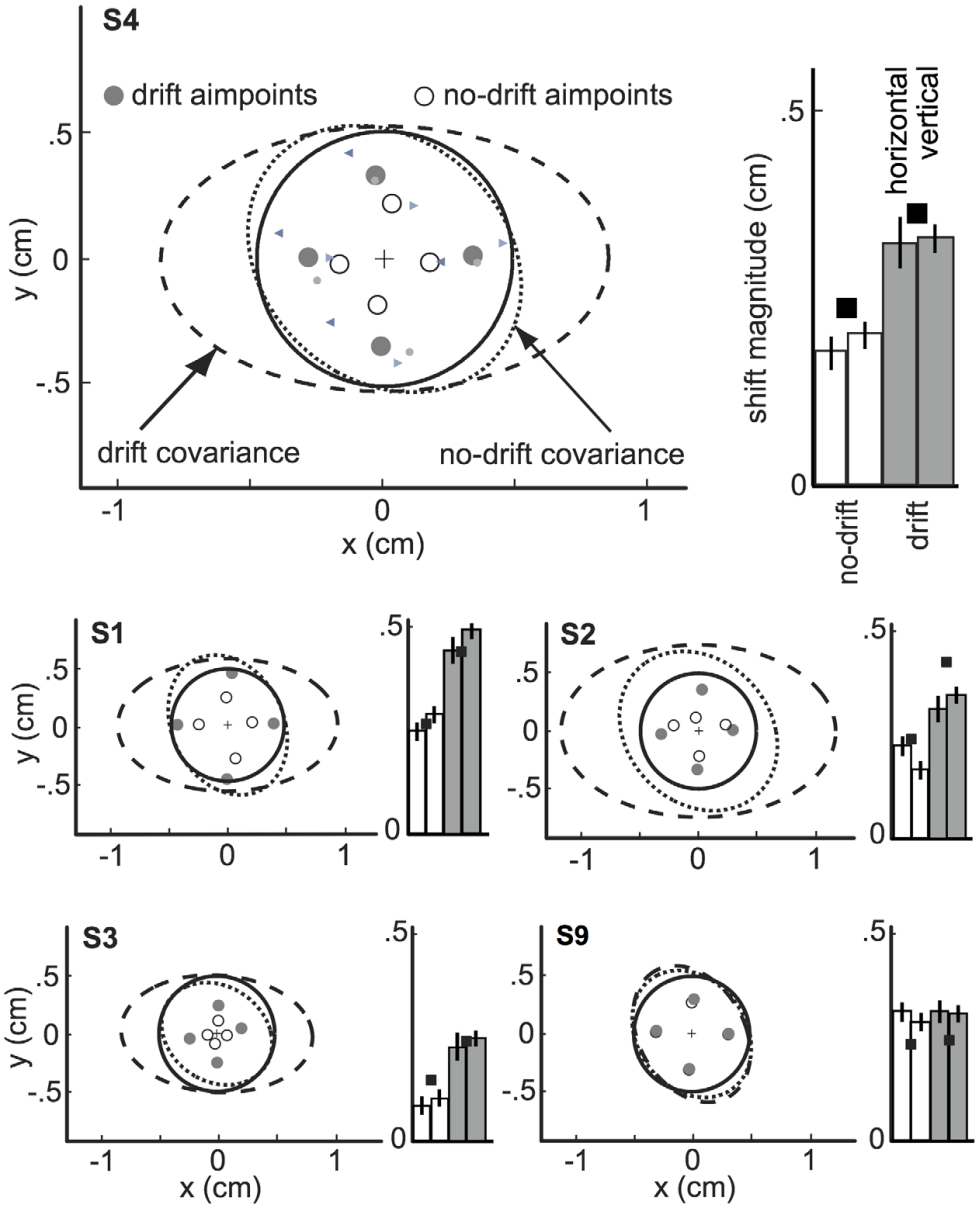
Mean endpoints and Δ_aim_ values. Top graph, left: The solid circle is the target region. Dotted and dashed ellipses are 2-SD covariance ellipses of endpoints in the no-drift and drift conditions, respectively. Open and filled circles are the mean endpoints in the no-drift and drift conditions, respectively. Mean endpoints on the left correspond to trials with penalty to the right of the target, endpoints below correspond to penalty above, etc. Small symbols correspond to the subsets of trials for the three drift conditions as in Fig. 2. Top graph, right: Bars indicate average Δ_aim_ values in the no-drift and drift conditions for horizontally and vertically displaced penalties. Black squares indicate predictions of the circular model (*M_c_*). All data for subject S4. Lower four graphs: same as above for S1, S2, S3, and S9.

Covariance ellipses calculated using data from zero-penalty blocks are shown for five subjects in Fig. 3 for the drift (dashed ellipses) and no-drift (dotted ellipses) conditions (see Supplementary Figure S2 for remaining subjects). Because the MFR perturbed reaches horizontally, ellipses derived from the drift session are elongated horizontally (subjects S1–S8), but not vertically. The ellipses of S9 show that this subject was insensitive to the MFR perturbation.

### Aimpoint planning

On each trial, a red penalty circle was located in one of four possible positions: above, below, to the left, or to the right of the target circle. When the imposed penalty was nonzero, subjects' mean endpoint locations (aimpoints) differed from the center of the target, and were located roughly along the target-penalty axis away from the penalty. This is in qualitative agreement with the movement plan that produces maximum expected gain (MEG, Fig. 1). In Fig. 3 and Supplementary Figure S2, aimpoints are plotted for each of the four penalty locations. Aimpoints to the left of the target center resulted from trials with the penalty on the right, and aimpoints below the target center from a penalty positioned above the target, etc. For subject S4, small symbols indicate mean endpoint locations for subsets of trials corresponding to each of the three drift types, showing that the MFR was as effective during nonzero-penalty blocks (Fig. 3 and Supplementary Figure S2) as it was in the zero-penalty blocks (Fig. 2). The bar graph shows the same aimpoints, measured as differences between aimpoints and the target center projected onto the target-penalty axis (Δ_aim_ values). Δ_aim_ values within a session were similar for all penalty locations, whether they were positioned horizontally or vertically relative to the target. In particular, Δ_aim_ measured during the MFR perturbation was nearly identical for all penalty locations, even though the drift perturbation only increased horizontal variance (S1–S8). Δ_aim_ was significantly larger in the drift relative to the no-drift session (*p*<.05) for these subjects regardless of penalty location.

### Simulated movement plans

We will compare our subjects' performance to the performance of simulated movement planners that maximize expected gain based on an internal model of motor noise using some or all of the covariance information available to our experimental subjects. Note that we do not model arm impedance because impedance control is not a strategy that appears to be engaged in response to visually-induced perturbation such as used by Wong and colleagues [17], and also during unpredictable MFR-induced visual-motor perturbation specifically [18]. Nor do we model corrections using joint-space coordinates, since (1) this would require an unparsimonious increase in model variables (joints×degrees of freedom), and (2) external-space frames of reference are used in preference over joint-space coordinate frames for predictive motor control [19].

We will compare the data to two simulated movement planners derived from two models of the internal representation of motor noise. Under the “anisotropic” model (*M_a_*), movement planners update an internal model based on the full covariance structure of the observed reach errors. Under the “circular” model (*M_c_*), movement planners update an internal model consisting of a single scalar estimate of motor variance. The latter model effectively assumes that motor variance is isotropic (i.e., circularly symmetric). In Fig. 2, the dotted and dashed ellipses are 2-SD contours of bivariate Gaussians fit to the data (left column), while in the right column those same data were fit with an isotropic Gaussian (averaging *x* and *y* variance). As expected, in the drift session (lower-right panel, dashed circle), the circular distribution underestimates the horizontal variance while overestimating the vertical variance when errors are in fact anisotropically distributed. Note that there are no free parameters in either the circular or anisotropic model (see Materials and Methods for details).

**Figure 4 pcbi-1000982-g004:**
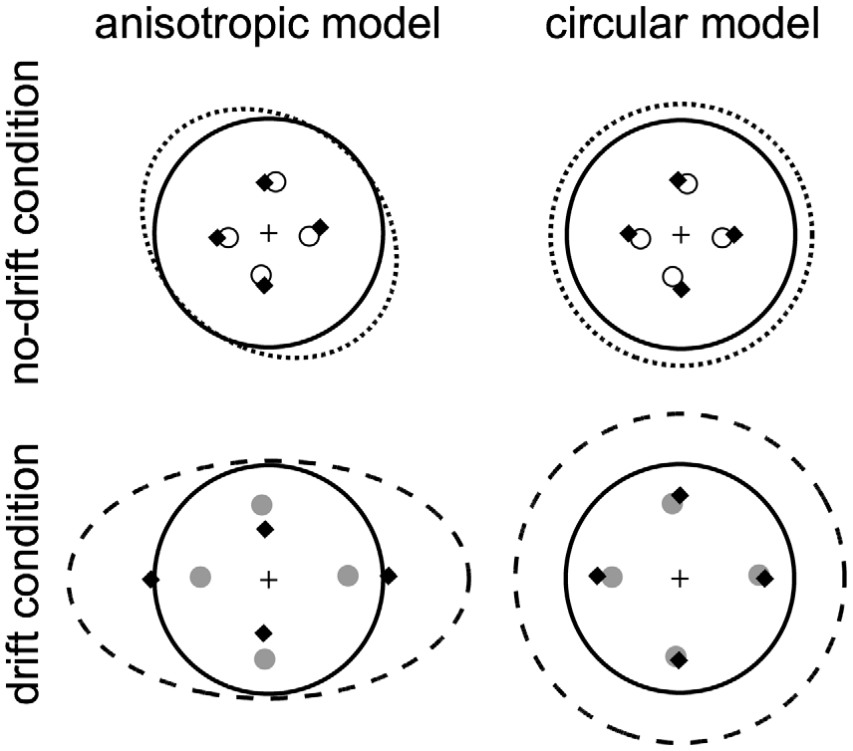
Ideal and observed aimpoints. Observed aimpoints (circles) are plotted along with MEG-predicted aimpoints (diamonds) for subject S4. Left column: MEG predictions based on the estimated covariance of endpoints in each condition. Right column: MEG predictions based on the fit of an isotropic Gaussian to the zero-penalty data. Dotted and dashed curves are identical to those in Fig. 2.

For each subject, we computed the ideal aimpoint maximizing expected gain for each of the four penalty locations for the two models described above. In Fig. 4 the full covariance ellipses (left column) and constrained circular fit (right column) to the zero-penalty data for subject S4 are plotted (organized as in Fig. 2). Mean endpoints (from Fig. 3, top panel) are plotted, along with the aimpoints predicted by models *M_a_* (left column) and *M_c_* (right column). Logically one might expect the endpoint distribution from the drift session to be a probability mixture of three Gaussians corresponding to the leftward, static and rightward drift trials. However, a qq-plot of the horizontal distribution of endpoints from the drift session to a Gaussian distribution indicated no patterned deviations (note the massive overlap of the distributions of the trials from the three intermixed drift conditions in Fig. 2). We model all endpoint distributions as bivariate Gaussian in computing predicted aimpoints for each model. For the no-drift sessions (top panels) where data covariance ellipses were nearly symmetrical, the two models predicted similar, nearly symmetrical aimpoints; both predicted the observed aimpoints well. For the drift sessions, the anisotropic model predicted too large a Δ_aim_ in the horizontal direction and too small a Δ_aim_ vertically. In contrast, the circular model predicted aimpoints closely corresponding to the observed data for all four target-penalty configurations.

**Figure 5:**
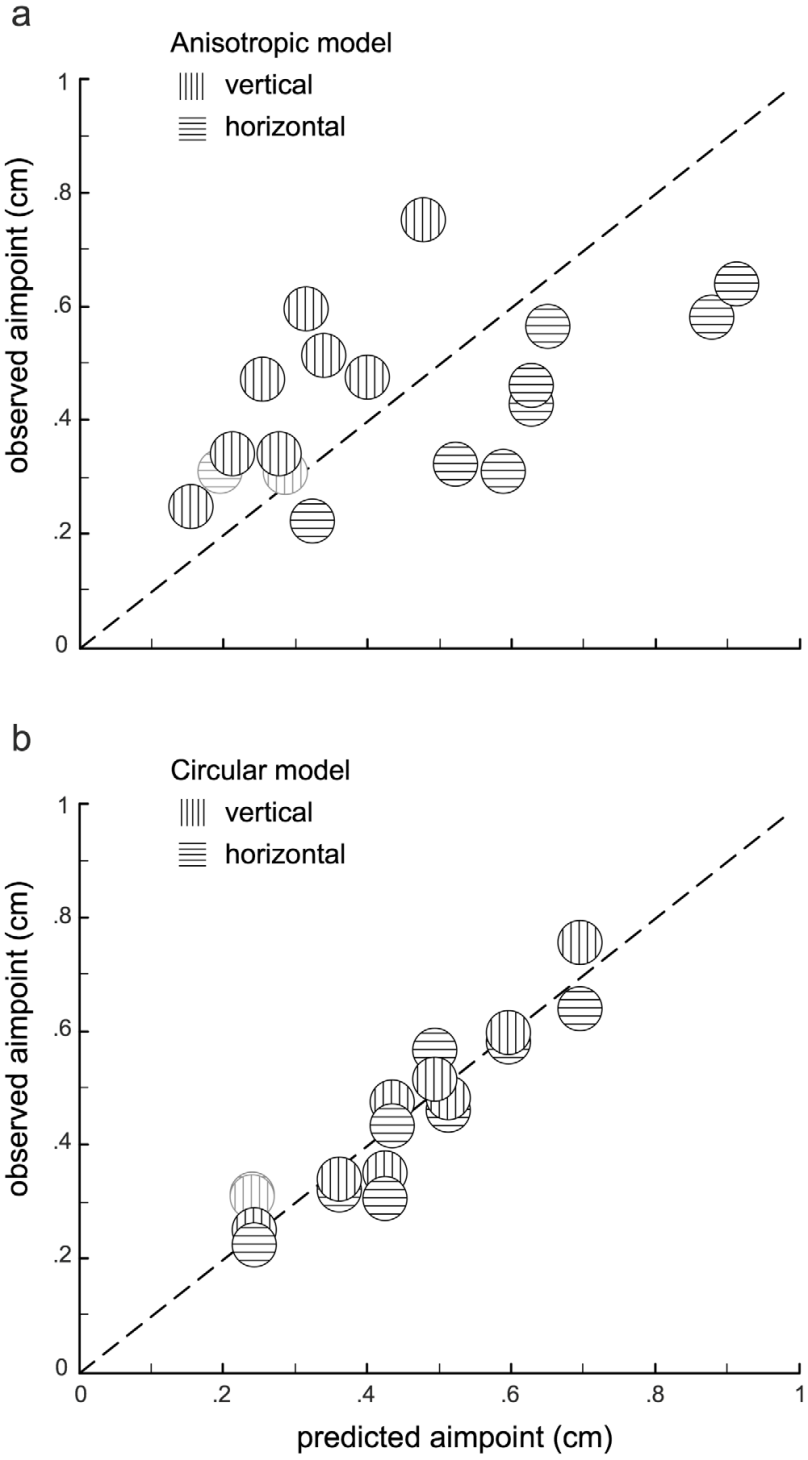
Model comparison. Δ_aim_ values for vertically and horizontally displaced penalties in the drift sessions are plotted as a function of MEG predictions based on an anisotropic estimate of endpoint variance (***a***, model *M*_a_) or variance based on the fit of a circular Gaussian to the zero-penalty data (***b***, model *M*_c_). In both panels, data for subject 5 (whose reaches were unaffected by visual drift) are shown in gray.

In Fig. 5 we plot Δ_aim_ (drift sessions only) relative to each set of Δ_aim_ values predicted by the two simulated movement planners (5*a*: *M_a_*; 5*b*: *M_c_*) detailed above. Observed aimpoints that closely approximate those predicted by *M_a_* or *M_c_* will fall near the identity line. Clearly, the simulated movement planner using a circular internal model of motor variance predicts the data better than the simulation using an anisotropic internal model of motor variance. The anisotropic model predicts too large a Δ_aim_ for horizontally displaced penalties, and too small a Δ_aim_ for vertically displaced penalties for all four subjects who were affected by the MFR perturbation.

Since subjects S2 and S3 received 2 points per hit, ideal Δ_aim_ values were in general smaller than those computed for the other subjects (who received 1 point per hit). Although not statistically significant, subjects' aimpoints vary according to this difference in reward function (Fig. 3) and closely track those predicted by model *M_c_* (Fig. 3, filled squares), which is consistent with previous work demonstrating that Δ_aim_ scales with differences in imposed reward [20].

Although the data are in qualitative correspondence with the predictions of the isotropic model, we next provide a direct quantitative comparison of the two models described above: *M*_a_ in which predicted aimpoints are those that maximize expected gain based on a general bivariate Gaussian (and therefore possibly anisotropic) error distribution, and *M*_c_ in which predicted mean endpoints are computed based on assuming covariance is isotropic (circular). We compare the two models by computing a measure of *evidence* [21]: 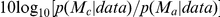, expressed in decibels. Evidence is therefore computed by comparing the data to predicted aimpoints derived from the two models [22]. These two probabilities are computed using the predicted aimpoints as well as the covariance matrix estimated from the zero-penalty data for each observer. Based on aimpoints computed from each subject's data, this calculation results in nearly 7000 dB of evidence in favor of *M*_c_, corresponding to odds in favor of *M*_c_ of 2.2×10^681^ ∶ 1.

We also computed a linear regression of observed Δ_aim_ values to Δ_aim_ values predicted by each of the two models. The best-fit slope and intercept to the data based on the predictions of the circular model (*α* = 02, *β* = 0.94 provide a much better approximation to an identity line (*α* = 0, *β* = 1) than the fit to the anisotropic model's data (*α* = 0.28, *β* = 0.34). This is consistent with the evidence calculation above.

Finally, for each subject we computed the averagαe, over the vertically and horizontally displaced penalties in the drift sessions, of the absolute value of the prediction error (observed minus predicted values of Δ_aim_) for the two models. The difference between these two values indicates the degree to which model *M*_c_ was more successful at predicting the observed data as compared to model *M*_a_. These values were significantly greater than zero (*t*(8) = 4.4, *p*<.01), also consistent with the evidence calculation.

Our analyses indicate strong support for the hypothesis that, in this task, the nervous system learns and takes into account the overall increase in motor noise affecting movement endpoints, but not the anisotropy of the noise covariance. This is consistent with the interpretation that observers learned that their motor variance was increased by the perturbation, but acted as if they only encoded the magnitudes of errors, not their directions.

The anomalous response of S9 to visual drift provides additional support for the idea that the CNS uses an internal model of its motor uncertainty in planning reaches that does not include information about the shape of the error distribution. S9 was the only subject unaffected by the MFR perturbation, and was also the only subject that maintained an identical movement strategy (identical aimpoints) during both the drift and no-drift sessions. Subject S9 certainly was aware of the visual drift, yet chose identical aimpoints over the two days. This suggests that observed Δ_aim_ in subjects affected by the MFR resulted from an updated internal model of motor variance rather than a simple reaction to suprathreshold visual motion per se. S9 also represents an extreme value on the continuum of individual responses to visual drift. S9 had no detectable response, whereas our other subjects exhibited nonzero, but nevertheless different, reflex responses to the visual drift. Regardless of each individual's MFR magnitude, all subjects demonstrate appropriate scaling for a circular internal model of motor variance in their Δ_aim_ data (Fig. 5*b*).

### Motor uncertainty

Table 1 summarizes the effectiveness of the unpredictable MFR perturbation for increasing horizontal movement variance based on the zero-penalty trials. Subject S9 did not respond to the MFR perturbation. For all other subjects, horizontal variance was greater than vertical variance in the drift condition, and horizontal variance was increased significantly in response to the MFR perturbation as compared to the no-drift condition. Finally, no subject's vertical variance differed between the drift and no-drift conditions, and in the no-drift conditions, no subject's horizontal and vertical variance differed significantly.

For each of the comparisons in Table 1, we also provide an evidence value using a Bayesian model comparison similar to that described above to compare *M_c_* and *M_a_*. For example, in the section of the table labeled “horizontal vs vertical (drift)” we compare a model in which the horizontal and vertical variance are assumed equal and a second model in which the horizontal variance is constrained to be greater than the vertical variance. Because only the horizontal and vertical dimensions are task-relevant, we assume the off-diagonal components of covariance matrices are negligible (i.e., zero correlation). In computing the evidence, we assumed a bivariate Gaussian distribution of endpoints and a Jeffreys prior [23] for motor error (for which 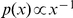). The results of the *F*-tests are consistent with these evidence calculations.

### Learning anisotropy

**Figure 6:**
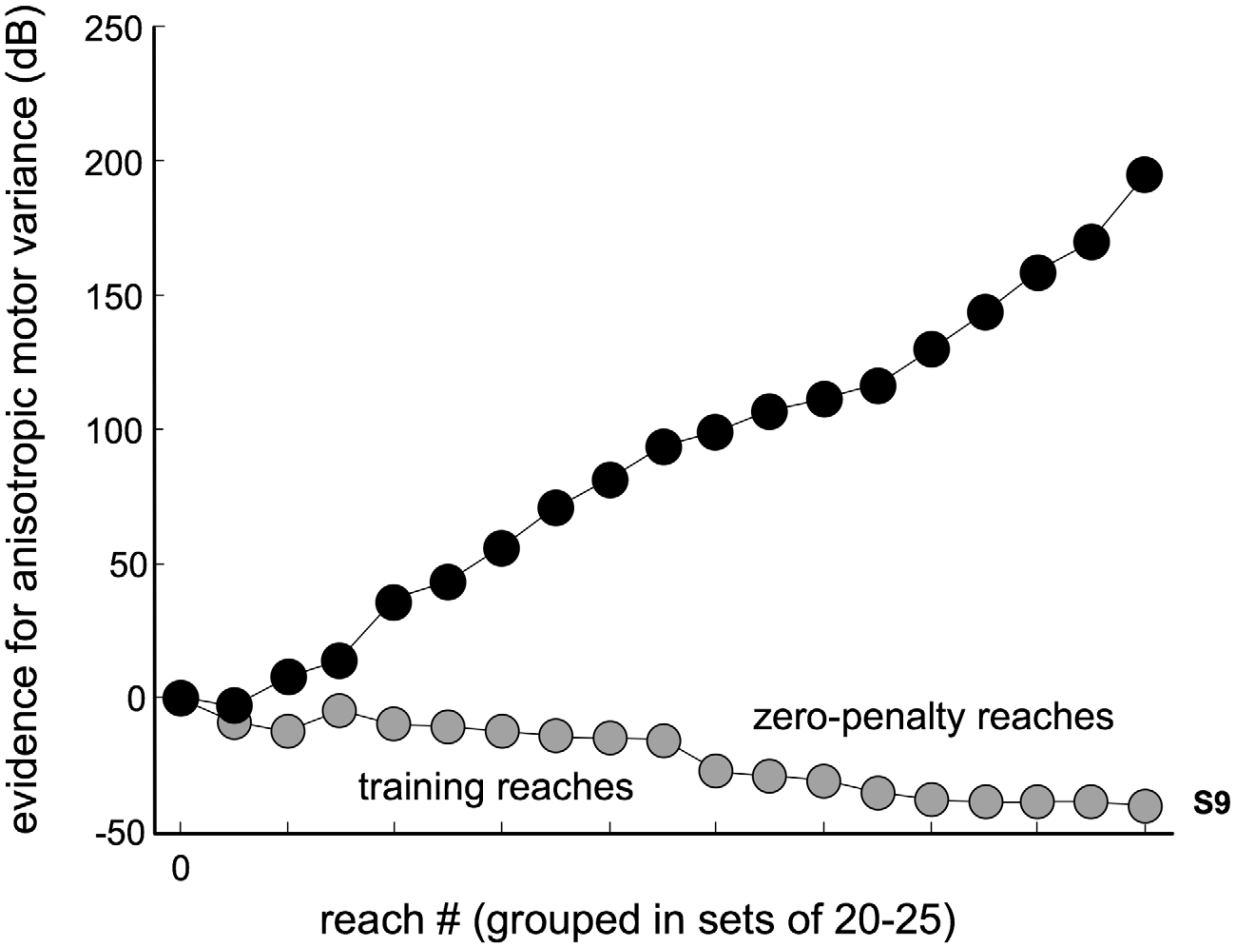
Evidence for anisotropic noise. During the drift session, reaches were perturbed by the manual following response. Evidence values quantify the information available to subjects signifying that the variance along the x-direction is greater than in the orthogonal direction (as opposed to being equal). Positive values are evidence in favor of the indicated anisotropy, and negative values are evidence in favor of equal variances. Each subject is plotted separately. By the end of the training portion of the drift session, the evidence overwhelmingly indicates that motor noise has become anisotropic (for S1–S8). Evidence in favor of this hypothesis continues to accumulate over the course of the session. Note that larger positive values indicate stronger evidence in favor of anisotropic variances, and not a larger anisotropy. That is, evidence values increase as more data consistent with the hypothesis of anisotropic variances becomes available (i.e., due to the increase of the size of the dataset with an increasing number of trials).

The anisotropic increase in motor error was introduced to each subject during a series of training reaches in which small crosshairs served as the target, and there was no penalty or payment bonus for missing or hitting the crosshairs. We next test whether subjects had enough information by the end of the training reaches to infer that movement uncertainty had increased anisotropically with a major axis oriented approximately horizontally, using an analogous evidence calculation to that just described. As shown in Fig. 6, the evidence in favor of inferring an anisotropic endpoint distribution increases from 0, prior to having made any reaches under the perturbation, to a substantial positive value for S1–S8 by the end of the training session; the evidence continues to increase throughout the drift session for these subjects. For subjects displaying an MFR, this calculation results in 127, 38, 270, 66, 34, 29, 70 and 76 dB at the end of the training reaches in favor of the model in which horizontal variance was constrained to be larger than vertical variance, corresponding to odds of at least about 800∶1. This indicates there was overwhelming evidence for these subjects to infer motor error anisotropy based on their training reaches before payoffs and penalties were introduced. In contrast, for subject S9 this value was −13.7 dB, indicating weaker, but nevertheless substantial evidence supporting isotropy (i.e., the opposite conclusion).

### Learning Δ_aim_ values

**Figure 7:**
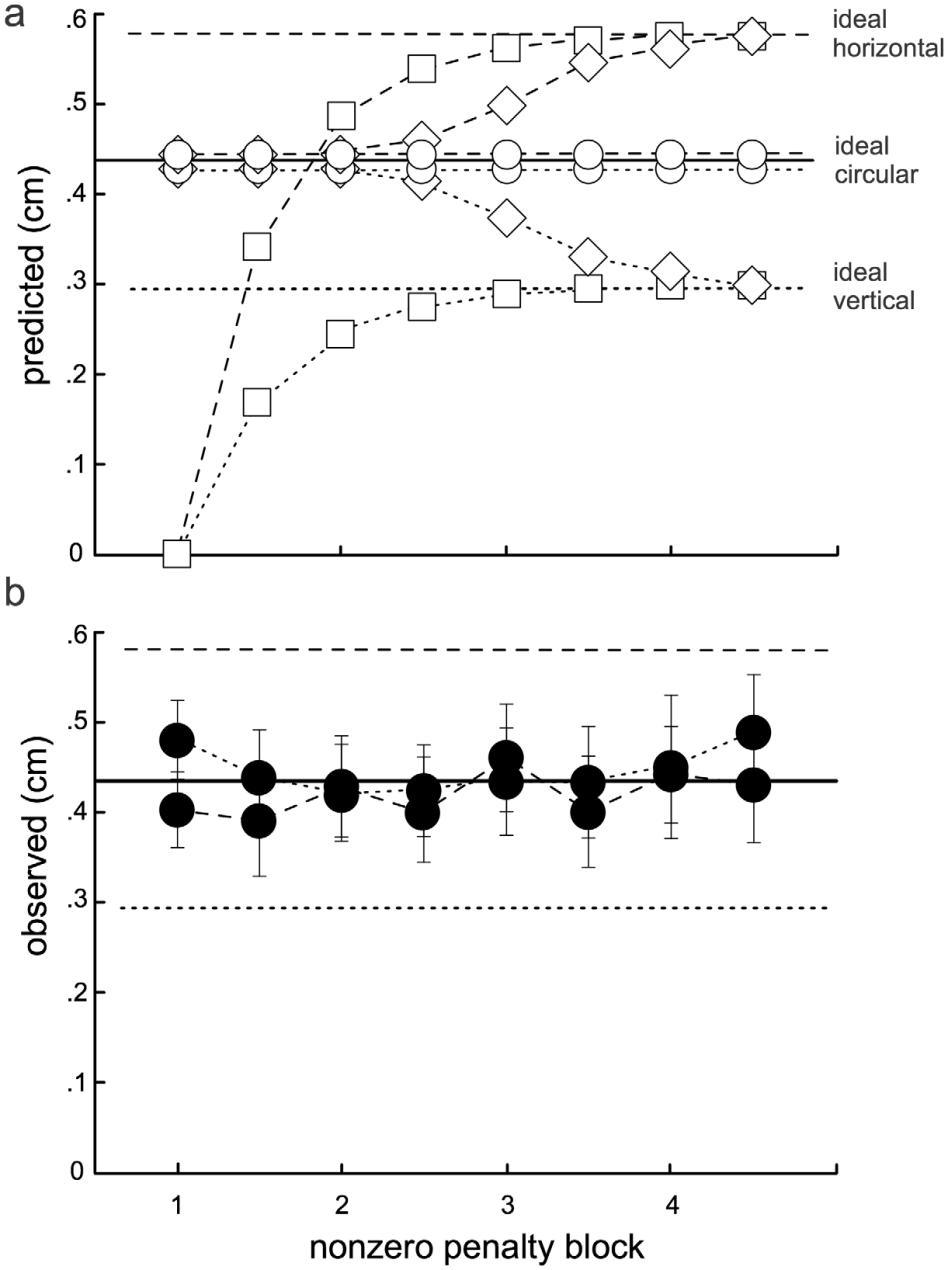
Time course of nonzero-penalty aimpoints. ***a***, Hypothetical data. Circles: aimpoints based on a stable internal model of endpoint variance using error magnitudes only. Diamonds: based on a process that has formed a circular model of endpoint variance as a transitory first step, but continues to update this model throughout training. Squares: based on a hill-climbing process responding solely to rewards and penalties. ***b***, Time course of observed Δ_aim_ values. Data are averaged over successive half-blocks of the 4 nonzero-penalty blocks (separating horizontally or vertically oriented target-penalty pairs), collapsed across subjects. Each subject was presented with 4 nonzero-penalty blocks and 2 zero-penalty blocks. These blocks occurred at different points in the experiment for different subjects. There is no hint that the two time courses diverge at any point during experiment.

Although Δ_aim_ values shown in Figs. 3–5 are consistent with the nervous system updating a strictly circular model of motor noise, these averages are computed over the entire drift session and may hide temporal structure that is inconsistent with such an hypothesis. Fig. 7*a* illustrates several hypothetical time courses of Δ_aim_ over the drift session. If movement planning during the drift sessions made use of a stable circular internal model of motor variance learned during the training reaches to crosshairs performed at the start of each session, Δ_aim_ values would be equal for vertically and horizontally oriented penalty regions at the value predicted by the circular model (open circles). If the circular variance is computed only as a transitory first step, and the internal model continues to be updated throughout training and testing, Δ_aim_ should gradually shift, and diverge for the two types of penalty location (diamonds) over the course of testing. Finally, if a hill-climbing strategy [14] were used for selecting aimpoints based only on rewards/penalties, then no internal model would be updated and one would predict a value of zero for Δ_aim_ resulting from the training session, and a gradual shift toward the respective optimal anisotropic values during the drift session (squares).

The observed time course, with Δ_aim_ averaged over successive half-blocks of the drift session, is shown in Fig. 7*b*. The results clearly indicate that subjects formed a stable, circular model of motor variance based on the 207 training reaches aiming at crosshairs, and show no evidence of adjusting that strategy over the 720 test reaches. Critically, note that the observed results are inconsistent with either of the two proposed alternatives, because both involve gradual shifts in Δ_aim_ over the course of testing, ultimately resulting in diverging Δ_aim_ values for horizontally and vertically positioned penalties that approximate the anisotropic model predictions.

## Discussion

Recent interest in probabilistic and neuroeconomic models of the nervous system has led to a new appreciation for the use of uncertainty information in perception and motor control [22], [24], [25], [26], [27], [28], [29], [30]. Here, we specifically perturb the motor system, introducing unpredictable reach errors resulting from unplanned motor torques. In a rapid pointing task under risk, we determine the statistical properties of movement error the CNS uses to correct for changing motor uncertainty. We ask whether compensation for changing uncertainty involves updating an internal model of the motor system, or simply incremental correction via a hill-climbing process. Although previous work has shown that the CNS can correct both a change in bias [1], [2], [3], [4], [31], [32], [33] or in outcome variance [15], [16], [22], [34], [35], this is the first demonstration that the CNS maintains an internal model of the motor system's error uncertainty as that uncertainty changes. Further, by using a perturbation that resulted in anisotropic motor noise, we were able to probe the structure of the internal model of motor uncertainty formed by the CNS. Remarkably, a constrained circular model of motor uncertainty was used for planning movements under risk during changing motor noise, despite the anisotropic distribution of endpoint errors.

Why would the CNS not encode additional statistics of the error distribution, beyond mean and variance? Certainly, for subjects S1–S8, the movement perturbation led to substantial error anisotropy (Fig. 3) that was readily detectable prior to selecting aimpoints for the various target-penalty configurations presented here (Fig. 6). Subjects completed over 200 perturbed training reaches at crosshairs before any reaches to target/penalty pairs were made, which provided sufficient data to all subjects about error anisotropy (Fig. 6). Despite over 200 reaches to crosshairs and over 700 reaches to target-penalty pairs during which learning could have occurred, there is no indication that reaches to horizontally and vertically oriented target-penalty pairs were planned differently at any point during the experiment (Fig. 7). Given that subjects learned the change in overall variance within approximately 200 reaches but failed to learn the anisotropy within 900 or more reaches, we believe the circular internal model acquired during training represents a stable response to the imposed anisotropic motor variance (Fig. 7). However, should the anisotropy be eventually learned over the course of days or months of practice, the fact that a circular internal model was formed and maintained over a behaviorally relevant period would continue to require explanation.

Consider, in this context, the proposal that the internal model of motor variance is updated according to Bayes' rule. Then, the observed lack of learning of the anisotropic component of the variance might be due to subjects' use of a prior distribution that favors isotropic descriptions of motor variance. Under this hypothesis, failure to learn anisotropy (despite accumulated evidence over training and experimental trials) would be due to a failure to overcome the isotropy bias of the prior. However, the evidence calculations shown in Fig. 6 indicate that the prior odds of an isotropic model relative to an anisotropic model would have needed to be about 10^20^∶1 for the average subject affected by the MFR, and in one case over 10^35^∶1, to account for the lack of learning displayed by these subjects. Given that in other circumstances (see below) the CNS is able to make use of information concerning anisotropic motor errors, we believe this represents an unrealistically concentrated prior distribution. Of course, because these prior odds estimates are derived values (based on Fig. 6), they assume Bayesian processing of (actual) motor errors across trials. Subjects clearly did not perform this idealized calculation, but our data cannot determine whether they failed to respond to anisotropy of errors due to errors in estimation of the magnitude of motor error in each trial (unlikely, since visual acuity is quite good), due to computational constraints, or for other reasons.

Another hypothesis that might be proposed to explain the isotropy of the updated noise model learned by our subjects is based on the fact that noise distributions *within* each of the three drift conditions are unchanged from the no-drift condition, and approximately isotropic. If the CNS began tracking 3 separate noise distributions in the drift session, the combination of these distributions would correctly predict the isotropy of the measured Δ_aim_ values. However, this hypothesis fails in its second prediction – that there should be no change in Δ_aim_ values between drift and no-drift sessions. Instead, Δ_aim_ values change between drift and no-drift sessions, and that change is quantitatively predicted by the magnitude of the overall noise distribution, not the average of the three drift-specific error distributions.

But was there a real, behaviorally relevant difference between using a circular vs. anisotropic internal model? In short, the answer is that subjects performed substantially worse (earned less money) by failing to use an anisotropic model of motor variance. We computed an efficiency measure for each condition and subject as the ratio of the number of points/trial expected based on observed aimpoints divided by the points/trial expected using the ideal anisotropic internal model. Efficiencies computed from the drift sessions were on average only 72% for subjects affected by the MFR. Efficiencies computed from no-drift sessions averaged 97%. In other words, subjects' earnings were substantially reduced by failing to take the anisotropic error distribution into account; and while it is always possible to suggest that a suboptimal result stems from the CNS using a broader cost function than used in a model, we see no theoretical reason why a second variance computation would be internally costly enough to offset a 25% drop in performance.

Subjects made optimal use of the overall variance of their motor errors, given the reward function imposed experimentally, and appeared to ignore the direction component of the error signal that was available to them. Does the motor system actually lack access to such information, or was it simply ignored? Many recent studies have demonstrated that motor learning involves multiple systems as seen, for example, in examining the time course of movement error correction to an imposed bias [31], [32], [36], [37], [38]. We suggest that the present result is consistent with the idea that the motor system encodes and represents the direction and extent of a reach independently [12]. Here, we are concerned with vector-coding of reach errors relative to the target, since for a given target the only component of the overall reach vector that differs from one reach to another corresponds to the direction and extent of the target-relative error vector. This manner of coding reach errors would allow the extent of motor errors to be used in updating an internal model of motor uncertainty, independent of direction information. Information concerning error direction is not lost, however, and is used for other purposes such as updating sensory-motor transforms in response to prism or other visual feedback disturbance [3], [15], or during adaptation to force perturbations [39], [40], [41].

While the nervous system's use of internal models has been demonstrated in many motor learning contexts [42], [43], [44], [45], in some instances evidence for the use of an internal model is lacking, and a simple error-corrective (‘hill-climbing’) process is a likely explanation. For example, impedance control, which can operate in tandem with predictive internal-model-based control mechanisms [e.g.], [ 46,47], has been shown to operate in this way [34]. As we describe above (Results: Learning Δ_aim_ values), use of a hill-climbing mechanism would have resulted in anisotropic compensations in the current study. Indeed, such anisotropic compensations have already been reported. Lametti and colleagues [34] demonstrated anisotropic changes in limb impedance when reaching to irregularly-shaped targets. They required subjects to make reaches within the plane containing an oriented target. Unlike in the present study, this allowed them to vary reach direction relative to target orientation. They showed that changing the orientation of an elliptical target in relation to the direction of the reach toward that target leads to compensatory changes in limb impedance. These changes in limb impedance result in anisotropic reach endpoints that, with practice, become aligned with the target orientation. The set of learned changes in limb impedance were consistent with a hill-climbing strategy that modifies impedance for each combination of reach direction and target orientation separately. Gepshtein and colleagues [16] also found anisotropic movement variance in a task in which subjects reached within the plane of the target toward an oriented target-penalty configuration; movement variance was larger along the direction of the reach. As in the Lametti et al. study, the target-penalty axis was either aligned with the reach direction or oriented relative to it (here, perpendicular to it). Aimpoints shifted further from the penalty for reaches aligned with the target-penalty axis than for reaches perpendicular to it. This compensation is similar to that found by Lametti et al. [34] in that it varied for each reach/target orientation, and is consistent with a hill-climbing strategy. Trommershäuser and colleagues did not provide analyses to test this hypothesis, however.

In the present study, we have investigated the properties of motor error signals that are used by the CNS to plan future movements. While others have looked at adaptation to bias in the face of stochastic perturbation [10], here we investigated what aspects of motor uncertainty are taken into account when planning movements. We found that movements with experimentally altered motor uncertainty stimulate updating of an internal model of the motor system, but only the overall variance, not the full anisotropic covariance matrix, was modeled. This was due to a failure to incorporate direction information from reach errors, in a manner consistent with current theories of motor planning and control [3], [12], [13].

## Materials and Methods

These experiments involved a reach paradigm that made use of a novel visuomotor perturbation. In our task, subjects earned a monetary reward by touching a small target circle within a short time window. Near each target was a small penalty circle. If subjects missed the target, they received no reward, but if they touched the penalty region they could incur monetary penalties. In the critical condition of the experiment, subjects made reaches that were unpredictably perturbed leftward, rightward, or not at all at the midpoint of the reach, using a visual-motor perturbation called the Manual Following Response (MFR). The MFR consists of large-field visual motion that perturbs the reach in the same direction as the motion [11], [48]. Because this unpredictable perturbation could not be corrected in the time it took subjects to complete these reaches [11], the perturbation had the effect of increasing motor uncertainty, but only in the horizontal dimension. Subjects could not plan in advance for any particular drift condition since these were randomly intermixed, nor could they compensate for the drift after it began due to the combination of time constraints imposed on the reach and the timing of drift onset (at the spatial midpoint of the reach).

With knowledge of the distribution of reach errors, it is possible to calculate the expected monetary gain associated with any potential aimpoint relative to a given target-penalty pair (Fig. 1). The peak of that gain landscape indicates the aimpoint resulting in maximum expected gain (MEG). Peaks are typically located away from the target center opposite the penalty region, by an amount that depends on the penalty location, penalty amount and the endpoint variance along the target-penalty axis. We will use evidence obtained from experimentally measured aimpoints both to infer the existence of an internal model of motor variance that is updated with changing motor variance, and to subsequently probe the structure of that internal model of motor variance. The latter is accomplished by comparing subjects' reaches to those of simulated movement planners that use either an anisotropic or a circular internal model of movement covariance.

### Subjects/ethics statement

Eight naive subjects and one author (S1) participated in this experiment. All subjects gave written informed consent before participation. Subjects were instructed to earn as many points as possible and were paid a bonus for the total points earned. Subjects were asked to complete two experimental sessions, over two days. No subject waited longer than one day between sessions. All procedures were approved by the NYU institutional review board.

### Apparatus

Subjects were seated in a dimly lit room with head positioned in a chin and forehead rest in front of a transparent polycarbonate screen mounted vertically just in front of a 21″ computer monitor (Sony Multiscan G500, 1920×1440 pixels, 60 Hz). The viewing distance was 42.5 cm. A Northern Digital Optotrak 3-d motion capture system (with two three-camera heads) was used to measure the position of the subject's right index finger. The camera heads were located above-left and above-right of the subject. Three infrared emitting diodes (IREDs) were located on a small (.75×7 cm) wing, bent 20 deg at the center, attached to a ring that was slid to the distal joint of the right index finger. Position data for each IRED were recorded at 200 Hz. The cameras were spatially calibrated before each experimental run, providing RMS accuracy of .1 mm within the volume immediately surrounding the subject and monitor apparatus (approximately 2 m^3^). A custom-made aluminum table secured the monitor and polycarbonate screen. A calibration screen was machined to accurately locate four IRED markers. A calibration procedure was repeated before each experimental run to put monitor display and Optotrak coordinates into register, based on the calibration screen and an additional IRED located at the front edge of the table. The distance from the starting point of the reach (near the front edge of the table) and the screen was 34.5 cm. The experiment was run using the Psychophysics Toolbox software [49], [50] and the Northern Digital API (for controlling the Optotrak) on a Pentium III Dell Precision workstation.

### Stimuli

Each session consisted of training and experimental trials. In training trials, subjects aimed at crosshairs (.4 cm width and height) whose locations were chosen randomly and uniformly within a 5×5 cm rectangle centered on the screen. In experimental trials, subjects aimed at a 1 cm diameter green circle next to a 1 cm diameter red circle (target and penalty regions, respectively). Hits within the target earned subjects one point. Hits within the penalty cost 0 or 5 points in separate blocks. The distance from center to center of each circle was always .75 cm. If the subject hit within the region of overlap between the target and penalty, the subject incurred both the gain associated with the target and the loss associated with the penalty. The center of the target region was chosen randomly and uniformly within a 5×5 cm rectangle centered on the screen, and corresponding penalty locations were chosen at one of 4 evenly spaced orientations relative to the target (above, below, to the left, or to the right).

There were two sessions: drift and no-drift. In the drift session, for both training and experimental trials, a large 0.05 cycle/deg vertical sinusoidal grating replaced the stimulus and filled the display at movement onset. During a reach, when the subject's finger traveled halfway to the screen, the grating either remained static or began to drift either rightward or leftward (speed: 20 deg/s); the three stimulus types occurred with equal probability. When the fingertip reached the screen, the grating disappeared and was replaced with the same stimulus that preceded the grating. In the no-drift session all trials were static, and were identical to the static trials from the drift session.

### Procedure

The two sessions (drift and no-drift) took place on separate days. Each session consisted of 3 blocks of training trials followed by 6 blocks of experimental trials. The order of the drift and no-drift sessions was counterbalanced across subjects.

#### All trials

A trial proceeded as follows: subjects brought their right index finger to the front edge of the aluminum table triggering the beginning of the trial. Next, the stimulus was displayed followed 50 ms later by a brief tone indicating that subjects could begin their reach when ready. Movement onset was defined as the moment the fingertip crossed a plane 3 mm in front of the table edge; the fingertip was required to reach the screen within 575 ms of movement onset. At movement onset, the grating appeared and, depending on the session and trial, remained static or drifted. A loud tone indicated a timeout (movement time greater than 575 ms). After reach termination, the stimulus reappeared in place of the grating along with a red dot (2 mm diam) indicating the reach endpoint.

#### Training trials

Subjects completed 3 blocks of 69 reaches to crosshairs to become comfortable with the task and the movement time constraint, and to learn their own (perturbed or unperturbed) motor noise. Subjects were instructed to try to hit the crosshairs as accurately as possible, without incurring a timeout.

#### Experimental trials

Following the training trials, subjects were introduced to the target and penalty regions, the gains and losses associated with them respectively, as well as the cost incurred for a timeout (7 points). Each day there were 6 blocks of 120 experimental trials. The cost for touching the penalty region was constant within each block and indicated to the subject beforehand. Subjects participated in 4 nonzero-penalty blocks in which the subject lost 5 points for touching the penalty circle, and 2 zero-penalty blocks in pseudo-randomized order such that the first three blocks always included one zero-penalty block, as did the final three blocks.

Subjects S1, S4–S9 earned 1 point for a hit within the target circle. They were also explicitly told the monetary value of the points ($.08 per point). Subjects S2 and S3 earned 2 points for a hit within the target circle and were paid $.04 per point (this variation in point structure allowed us to verify that responses to the drift perturbation scaled with reward). Auditory feedback was provided for hits within the target region and/or penalty region, along with a display of the points earned or lost in that trial. If subjects did not complete the reach within 575 ms, auditory feedback was provided with the message “TIME OUT, −7”, regardless of the landing position of the fingertip. No subject incurred greater than 23 timeouts in the 720 trials of each session.

### Data collection

Before each experimental session, subjects (fitted with IREDs) placed their right index finger (pointing finger) at a calibration location on the screen while the Optotrak recorded the locations of the three IREDs on the finger 150 times. For each set of measurements, we computed the vectors from the central IRED to the two others, the cross product of those vectors (thus defining a coordinate system centered on the central IRED), and the vector from the central IRED to the known calibration location. We determined the best linear transformation that converted the three vectors defining the coordinate frame into the vector indicating fingertip location. On each trial we recorded the 3-d positions of the IREDs at a rate of 200 Hz and converted them into fingertip location using this transformation. Trials in which the subject failed to reach the screen within 575 ms of movement onset were excluded from further analysis.

### Data analysis

The focus of our analysis was the finger landing point on the screen relative to the actual target location. Thus, endpoint data were transformed from Optotrak to screen coordinates. Data were analyzed separately for each subject.

We computed the mean endpoint location for each target-penalty configuration separately for each subject. As would be expected, in zero-penalty blocks mean endpoint location (i.e. aimpoint) never differed from the center of the target (<2 mm bias for all subjects). We computed the sample covariance of the bivariate distribution of endpoints from the zero-penalty experimental trials (for each subject, in each session). In addition, we fit an isotropic (circular) bivariate Gaussian to these same data by pooling the variance in *x* and *y* 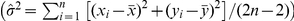.

For each variance model, we then calculated the expected gain for each of a finely spaced grid of potential aimpoints over the target region for the nonzero-penalty conditions. Expected gain was computed as follows:
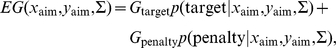
where 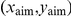 and **Σ** are the aimpoint and covariance under consideration, the *G*'s are the gains associated with landing in the target or penalty, and the probabilities of landing in each are computed by integrating the bivariate Gaussian defined by 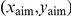 and **Σ** over the target or penalty region. The MEG aimpoint was defined as the aimpoint within the grid that maximized EG (Fig. 1). We assumed the covariance did not differ between zero- and nonzero-penalty blocks. Analyses based on the ratio of sample variances between zero- and nonzero-penalty blocks for each subject and penalty location support this assumption (all *p*-values>.01, 0.06<F<1.64 before correction for multiple comparisons).

### Statistical analysis

#### Effectiveness of the movement perturbation

When the principal axes of the covariance ellipses (Figs. 2–4) are nearly parallel to the *x*- and *y*-axes as they were during the drift sessions, only the horizontal variance matters for maximizing gain in response to the horizontally displaced penalties, and likewise only the vertical variance matters for the vertically displaced penalties. We compared the horizontal to the vertical marginal variances as a test of task-relevant anisotropy and also compared each marginal variance across the drift and no-drift sessions, separately for each subject. This was done by determining whether the ratio of sample variances was significantly greater than one (using an *F* distribution, Table 1).

#### Δ_aim_ values

We refer to the difference between the aimpoint and the target center measured along the target-penalty axis as Δ_aim_ (where positive values indicate aimpoints on the side of the target opposite the penalty region). In Fig. 3, the error bars on the bar graphs of Δ_aim_ value represent ±1 standard error of the mean across trials. When mean endpoints are compared, t-tests were used.

#### Model selection

We compared two models of aimpoint selection in the drift session. For model *M*_a_ (anisotropic error model), MEG aimpoints were computed based on Gaussian errors with covariance matrix 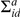 estimated using subject *i*'s zero-penalty data from the drift session (Fig. 4, lower-left panel). Predicted aimpoints for model *M*_c_ (circular error model) were computed similarly, except a circular (isotropic) Gaussian error model with covariance matrix 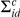 was used (Fig. 4, lower-right panel). For each subject *i* and penalty offset condition *j* (corresponding to penalty regions located above, below, to the left and right of the target), model *M*_a_ predicted aimpoint location 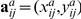 and similarly for model *M*_c_. Corresponding calculations were made using the data in the no-drift sessions based on estimated covariance matrices 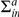 and 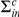 of the no-drift data (Fig. 4, upper panels).

For the drift data, we calculated the likelihood of model *M*_m_ (where *m* = *a* or *c*) for each subject *i*'s data *D_i_* as
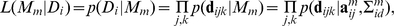
where 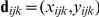 is the endpoint of the *i* ^th^ subject on the *k* ^th^ trial in condition *j*. To calculate this probability, we assume only a bivariate endpoint distribution with finite covariance matrix (which, based on maximum-entropy arguments, defines a bivariate Gaussian distribution), using our best estimate of the covariance matrix for each subject in the drift session, 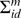:
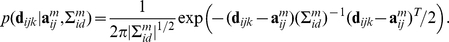
Finally, we calculated the evidence supporting the circular model *M*_c_ as compared to the anisotropic model *M*_a_ as
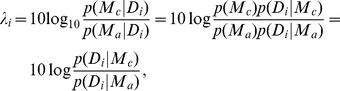
where we use a uniform prior probability over models to represent our lack of a prior preference for one model or the other. The resulting evidence value is in units of decibels of evidence [21] as we have used in previous work [22].

## Supporting Information

Figure S1 Best-fit 2D variance ellipses for subjects not shown in Figure 2.
(2.06 MB EPS)

Figure S2 Additional subjects not contained in Figure 3.
(0.37 MB EPS)
